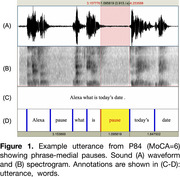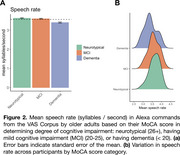# Speech rate as a biomarker of cognitive impairment in technology‐directed tasks

**DOI:** 10.1002/alz70856_098705

**Published:** 2025-12-24

**Authors:** Michelle Cohn, Alyssa Weakley, Alyssa M Lanzi

**Affiliations:** ^1^ University of California, Davis, Davis, CA, USA; ^2^ University of California, Davis, Sacramento, CA, USA; ^3^ University of Delaware, Newark, DE, USA

## Abstract

**Background:**

Millions of people now regularly talk to voice technology (e.g., Siri, Alexa), which has sparked an interest in detecting a person's cognitive status from these interactions. While a slower speech rate has emerged as a potential behavioral biomarker of cognitive impairment, the vast majority of prior studies have examined human‐directed tasks (e.g., describing a picture to an experimenter). However, our related work has shown that people slow their rate when talking to technology, compared to a real person. The current study investigates whether speech rate is a reliable biomarker of cognitive impairment in technology‐directed tasks.

**Methods:**

We analyzed recordings from the Voice Assistant System (VAS) corpus (Liang et al., 2022) wherein participants read commands to an Alexa voice assistant (e.g., “Alexa, what is today's date?”)(see Figure 1). Participants (*n* = 89, ages 65‐94, 46 women, 44 men) varied in MoCA score: as neurotypical (*n* = 30; MoCA 26+), having mild cognitive impairment (*n* = 35; MoCA 20‐25), or having dementia (*n* = 24; MoCA < 20). We measured utterance‐level speech rate (mean number of syllables per second) using a Praat script and modeled it with a mixed effects regression. Predictors included MoCA score, Trial, Gender, Age (and all possible interactions), as well as Geriatric Depression Scale, Generalized Anxiety Score, and Previous Voice Technology Experience (yes, no). Random effects included by‐Participant and by‐Command random intercepts and by‐Participant random slopes for Trial Number.

**Results:**

Individuals with higher MoCA scores produced their Alexa‐directed commands with a faster speech rate [Coef = 0.02, *p* < 0.01] (see Figure 2A). There was also an effect of Gender: women tended to produce commands at a faster rate than men [Coef = 0.07, *p* < 0.05]. There were no other significant effects. However, there was considerable variation across participants in their speech rate for commands, leading to overlapping distributions for neurotypical adults and individuals with mild cognitive impairment and dementia (see Figure 2B).

**Conclusions:**

This study suggests that a reduced speech rate is associated with cognitive impairment in technology‐directed commands, indicating that a slower rate might be a sensitive, albeit nonspecific, behavioral biomarker.